# Prenatal diagnosis of a silver-russell syndrome caused by 11p15 duplication and pedigree analysis

**DOI:** 10.3389/fgene.2024.1465521

**Published:** 2024-12-13

**Authors:** Shurong Hong, Hua Wei, Xueyi Zhuang, Weirong Huang, Yu Zhang

**Affiliations:** ^1^ Department of Molecular Genetic Center, Zhangzhou Municipal Hospital Affiliated to Fujian Medical University, Zhangzhou, China; ^2^ Department of Obstetrics, Zhangzhou Municipal Hospital Affiliated to Fujian Medical University, Zhangzhou, China

**Keywords:** SRS, 11p15.4–15.5 duplication, IC2, prenatal diagnosis, phenotype

## Abstract

**Introduction:**

Silver-Russell syndrome (SRS) is an imprinting disorder characterized by intrauterine and postnatal growth retardation. The pathogenic alterations and phenotypes are heterogeneous.

**Methods:**

Here, we present a rare pedigree of duplications with different methylation patterns in 11p15.5, which caused SRS or a normal phenotype across three generations.

**Results:**

Duplications of maternal IC2 (copy number of 3) with enhanced methylation (methylation index of 0.62) resulted in typical SRS.

**Conclusion:**

The result added to the complexity of the molecular genetics of SRS.

## 1 Introduction

Genomic imprinting is an epigenetic modification that enables the expression of certain genes in a parent-of-origin manner (paternal or maternal origin). The proper setting of imprinting marks plays an important role in intrauterine and postnatal growth (Eggermann et al., 2021). Aberrant imprinting signatures are associated with imprinting disorders (ImpDis), resulting in growth disturbance as a major clinical hallmark (Eggermann, 2024). Imprinted genes cluster at specific chromosomal regions called imprinting control regions (ICs), and their expression is commonly regulated by altered methylations, either by loss or gain of methylation (Elhamamsy, 2017). The changes in the coding sequences or CNVs could also affect the expressed allele (Mackay et al., 2022). The 11p15.5 chromosomal region has two important ICs, IC1 and IC2. IC1 includes the *H19/IGF2* which is methylated on the paternal allele. IC2 harbors *CDKN1C/KCNQ1OT1* which is maternally methylated (Eggermann et al., 2016). Opposite epigenetic and genomic disturbances in this chromosomal region contribute to two ImpDis known as Silver-Russell syndrome (SRS, OMIM 180860) and Beckwith–Wiedemann syndrome (BWS, OMIM 130650).

SRS is characterized by prenatal and postnatal growth restriction, relative macrocephaly, body asymmetry and characteristic facial features (Ishida, 2016). The patients usually have feeding difficulties, endocrine and metabolic abnormalities since infancy, which not only lead to short stature but also cause nutritional disorders that might affect neurodevelopment (Russell, 1954). As opposed to SRS, BWS is characterized by macroglossia, pre- or postnatal macrosomia, abdominal wall defects, and an increased risk for embryonal tumors (Ibrahim et al., 2014). Typically, duplications of the maternal allele at chromosome 11p15.5 or loss of methylation (LOM) of IC1 enhancing the expression of *IGF2* result in SRS, whereas duplications of the paternal allele or gain of methylation (GOM) of IC1 are associated with BWS (Gicquel et al., 2005; Kraft et al., 2019). Although the two diseases are considered to be related to genetic anomalies on the same chromosome, the pathogenic alterations and phenotypes are heterogeneous.

Accurate diagnosis of SRS is a challenge since postnatal growth overlap with other diseases, and facial features ease with age. The estimated incidence of SRS was between 1/75,000 and 1/100,000 worldwide (Eggermann et al., 2015). However, the actual incidence of SRS may be underestimated, as a large part of the affected population is not diagnosed. Yakoreva et al. published a nationwide incidence as high as 1/15,866 in an epidemiological study (Yakoreva et al., 2019). The Netchine-Harbison clinical score system (NH-CSS) recommends the clinical diagnostic criteria for SRS (Wakeling et al., 2017). In 65% of patients with SRS, an underlying molecular aberration can be detected (Lokulo-Sodipe et al., 2020). About 50% of SRS patients have LOM of IC1 on chromosome 11p15.5, and about 5%–10% of patients carry maternal uniparental disomy (UPD) of chromosome 7 (UPD (7) mat) (Lokulo-Sodipe et al., 2020; Netchine et al., 2007). In addition, UPD (14) mat was also reported in SRS (Ioannides et al., 2014). In the remaining 30%–40% of patients, the molecular etiology remains unclear. Patients diagnosed timely might partially benefit from growth hormone (GH) replacement, and they require ongoing monitoring for medical issues into adulthood. Early interventions such as enriched nutrition and GH treatment can improve final height and neurological outcomes (Lokulo-Sodipe et al., 2022). In the present study, we reported a rare SRS case with 11p15.4–15.5 duplication, which was transmitted across three generations and diagnosed prenatally for better prenatal consultation and postnatal management. Our findings might add to the complexity of the molecular etiology of SRS.

## 2 Materials and methods

### 2.1 Samples

**FIGURE 1:**
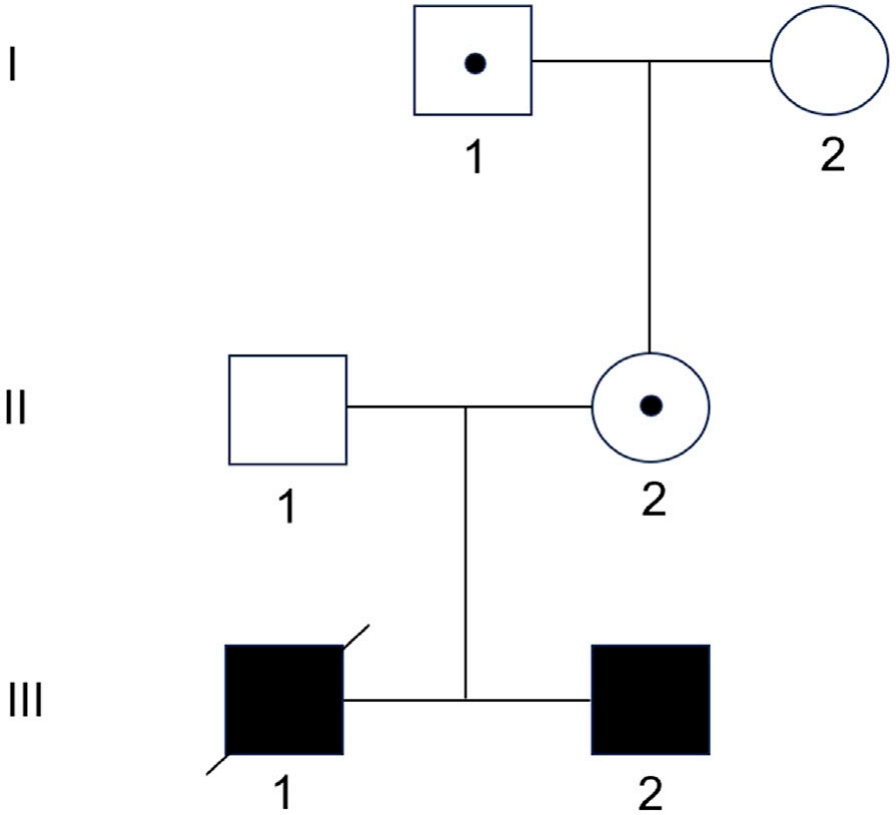
Family pedigree. The grandfather (I-1) and mother (II-2) are healthy carriers of the 11p15.5 duplication. The mother has transmitted it to both sons.

Amniotic fluid shed cells (III), peripheral blood samples from the parents (II) and maternal grandparents (I), and umbilical cord blood samples from the propositus (III-1) were obtained for molecular analysis (Figure 1). Written informed consent was obtained from the parents. The study was approved by the Institutional Review Board (IRB) of Zhangzhou Affiliated Hospital of Fujian Medical University (2022LWB268).

## 3 Testing methods

DNA was extracted from the samples and chromosome microarray analysis (CMA) was performed using the Affymetrix CytoScan 750K Array following the manufacturer’s instructions.

## 4 Methylation-specific multiplex ligation dependent probe amplification (MS-MLPA)

MLPA was performed with the SALSA MS-MLPA Probemix ME028-C1 BWS/RSS (MRC-Holland, Amsterdam, Netherlands), according to the manufacturer’s protocol. The kit contains probes evaluating the presence of deletions and duplications in the 11p15.5 genomic region, which includes the following genes: *H19, IGF2, KCNQ1, KCNQ1OT1, CDKN1C*; and methylation status of imprinting centers, IC1 and IC2. PCR products were analyzed using ABI 3130xl Genetic Analyzer (Applied Biosystems, USA) and Genemark v.1.51 software.

### 4.1 Clinical case presentation and results

#### 4.1.1 Parental phenotype

A pregnant woman, 27 years old, height 156 cm, with a spouse height of 178 cm, both with normal phenotypes, and in a non-consanguineous marriage.

#### 4.1.2 First pregnancy and propositus

Fetal ultrasound at 30 + 1 gestational weeks (based on the mother’s menstrual cycle) showed fetal growth restriction (FGR), with an estimated gestational age (GA) of 26+2 weeks. Amniocentesis was performed for prenatal diagnosis. Fetal CMA showed a 1.04 Mb duplication in 11p15.4–15.5 (arr [GRCh37]11p15.5p15.4 (2,154624_3191365)×3). The mother’s CMA showed a 1.14 Mb duplication in 11p15.4–15.5 (arr [GRCh37]11p15.5p15.4 (2015691_3150687)×3) which mostly overlapped with the fetal segment. Comparison analysis showed the same genes included in both fetal and maternal duplications, indicating an unknown clinical significance of copy number variation (CNV). The mother chose to terminate the pregnancy at 32 gestational weeks. The abortus weighed 900 g. Further whole exome sequencing revealed three variants of uncertain pathogenicity (*CBL* gene c.1766C>T: p.S589L, heterozygous, maternal origin; *POLA1* gene c.4147–57C>T, hemizygous, maternal origin; *CHD4* gene c.4627C>G: p.P1543A, heterozygous, paternal origin).

#### 4.1.3 Second pregnancy and survivor

**FIGURE 2:**
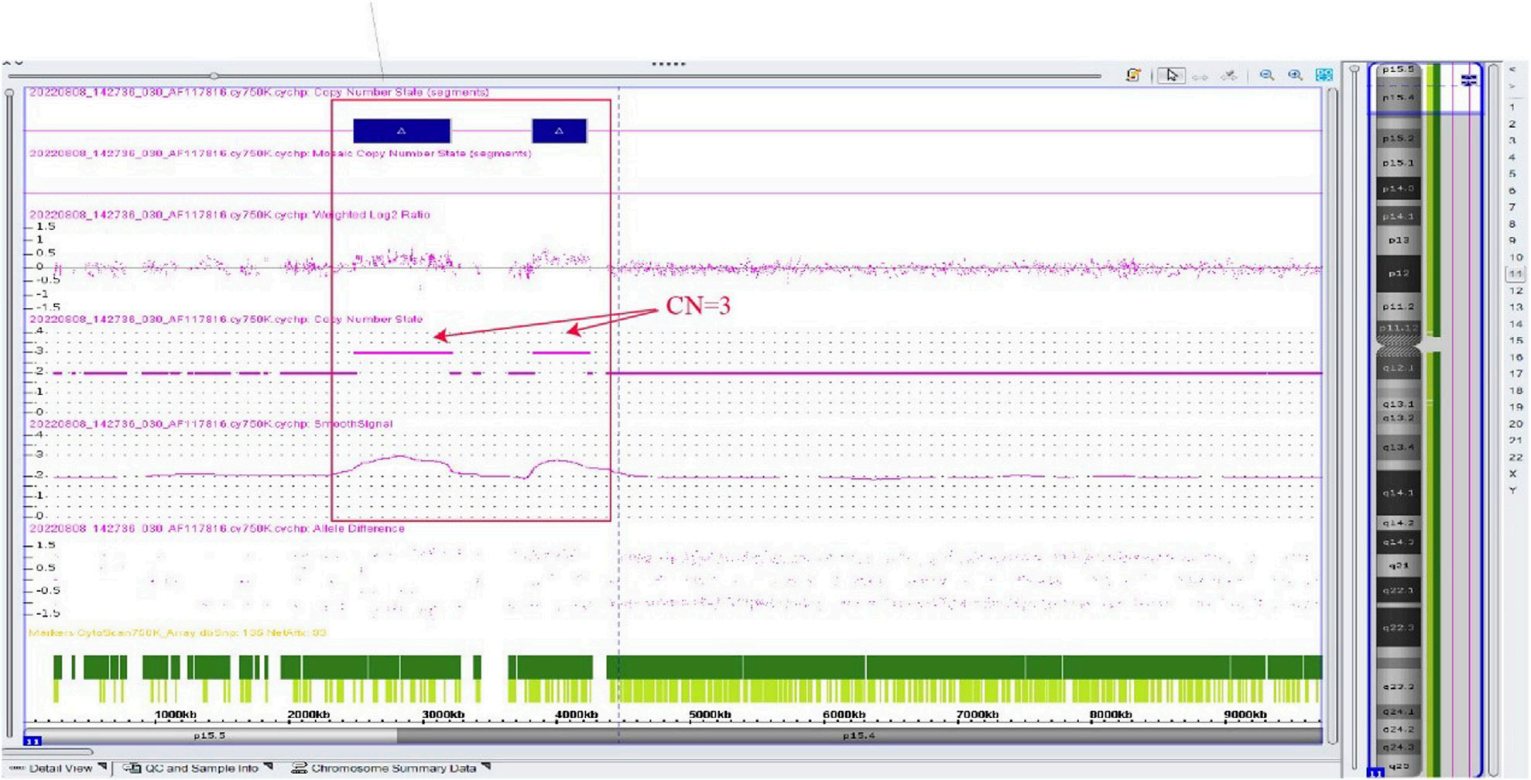
Chromosome microarray result of the fetus in the second pregnancy, showing two repeated segments. arr [GRCh37]11p15.5p15.4 (2,480,224_3,196,694)×3, arr [GRCh37]11p15.5p15.4 (3,813,457_4,222,358) × 3.

**FIGURE 3:**
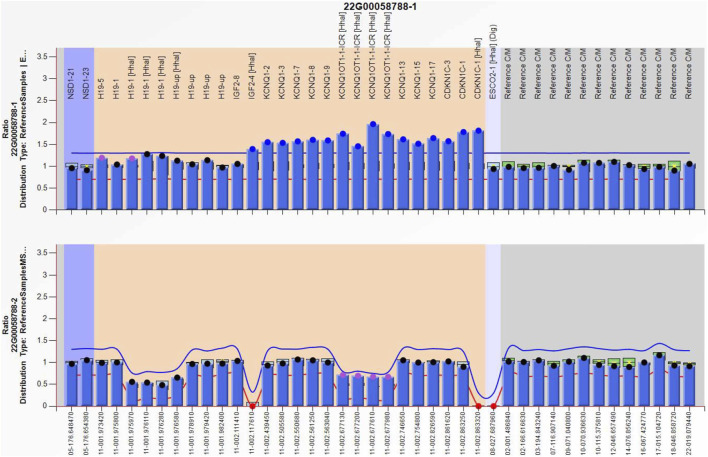
Methylation-specific multiplex ligation-dependent probe amplification result of the fetus in the second pregnancy, showing IC2 repeated involving *KCNQ1OT1* and *CDKN1C* (top), and IC2 methylation was enhanced (bottom), IC1 was normal.

**FIGURE 4:**
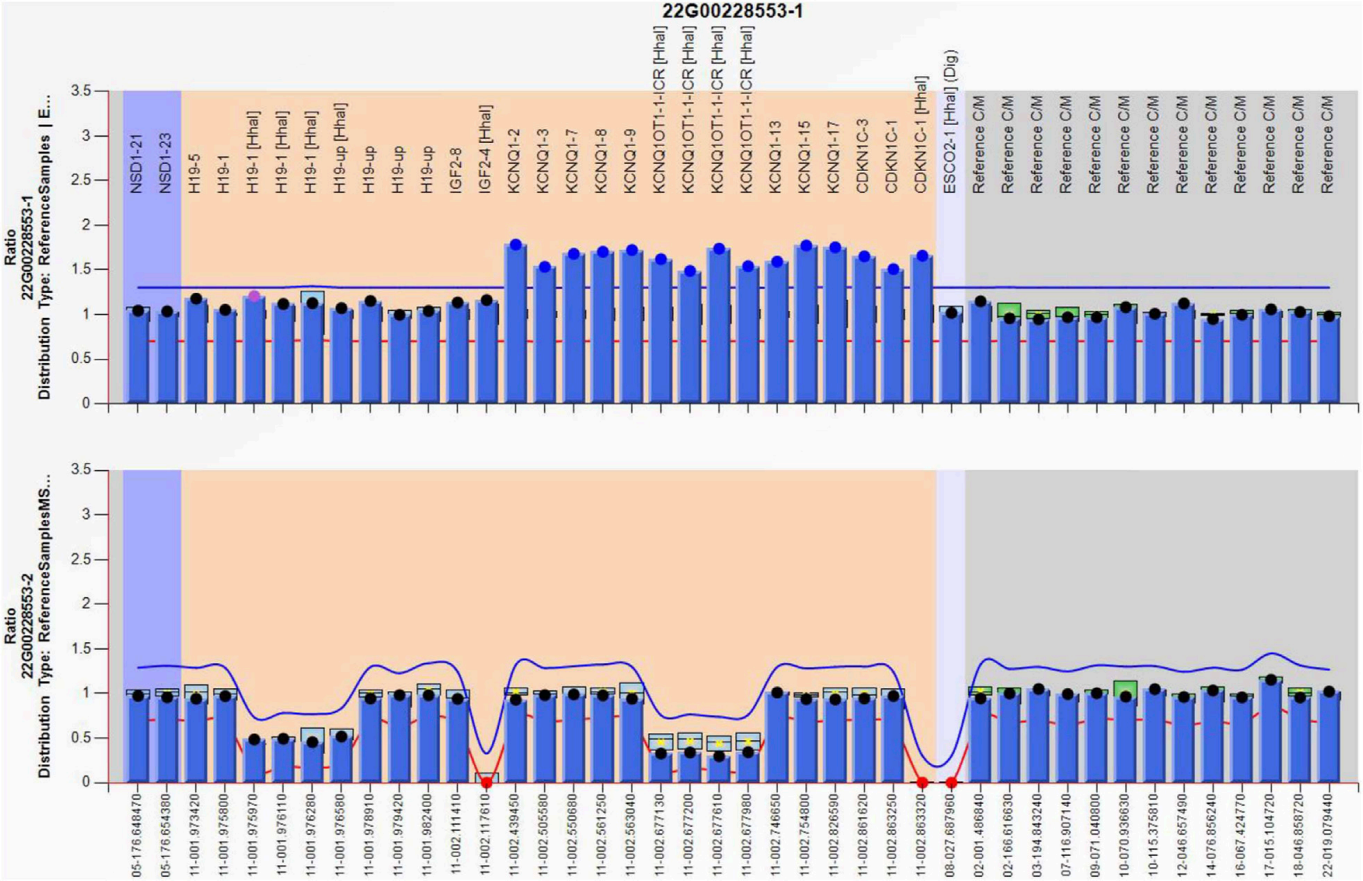
Methylation-specific multiplex ligation-dependent probe amplification result of the mother, showing IC2 repeated involving *KCNQ1OT1* and *CDKN1C* (top), and IC2 methylation was low (bottom), IC1 was normal.

**FIGURE 5:**
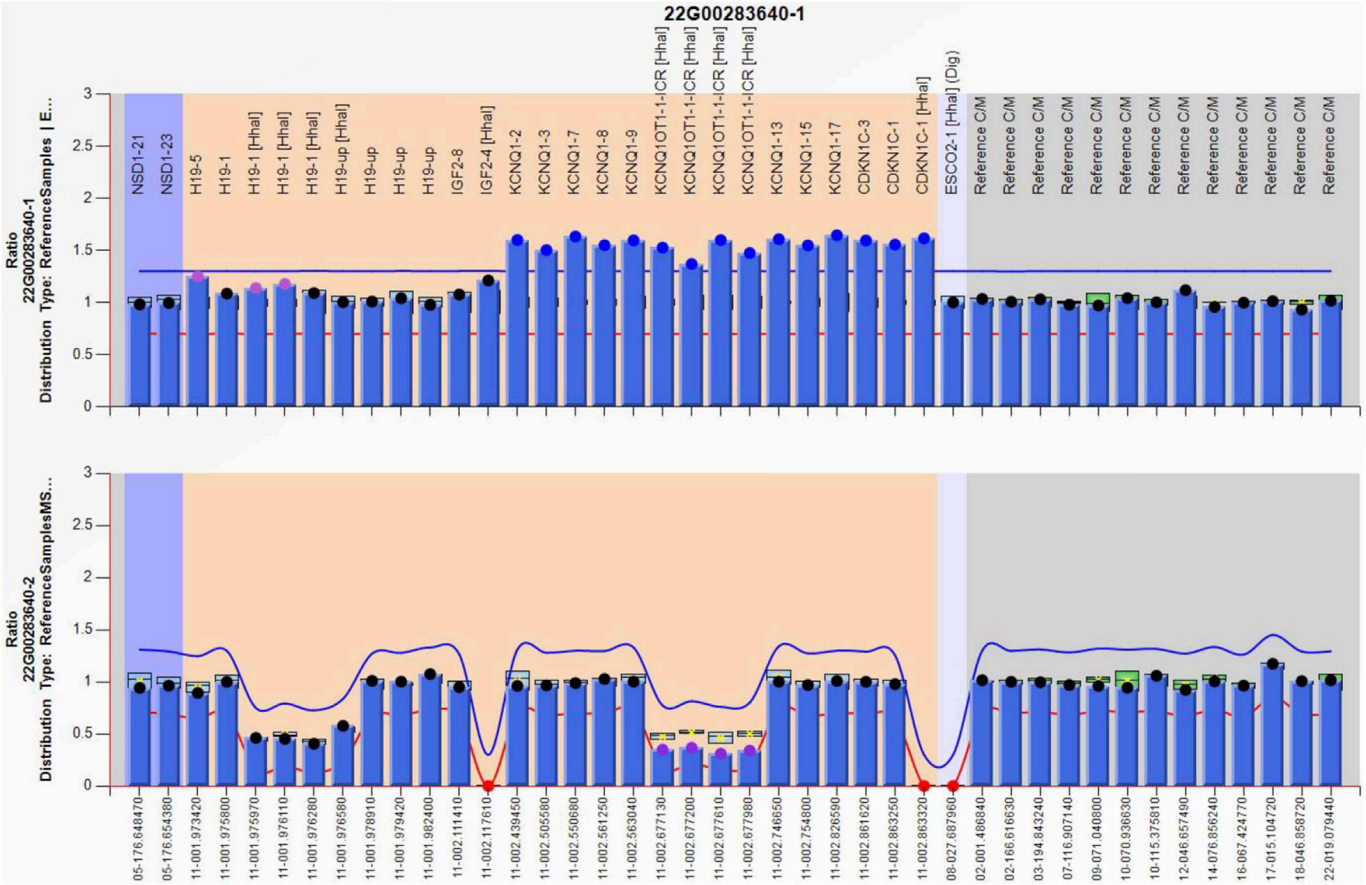
Methylation-specific multiplex ligation-dependent probe amplification result of the grandfather, showing IC2 repeated involving *KCNQ1OT1* and *CDKN1C* (top), and IC2 methylation was low (bottom), IC1 was normal.

**FIGURE 6:**
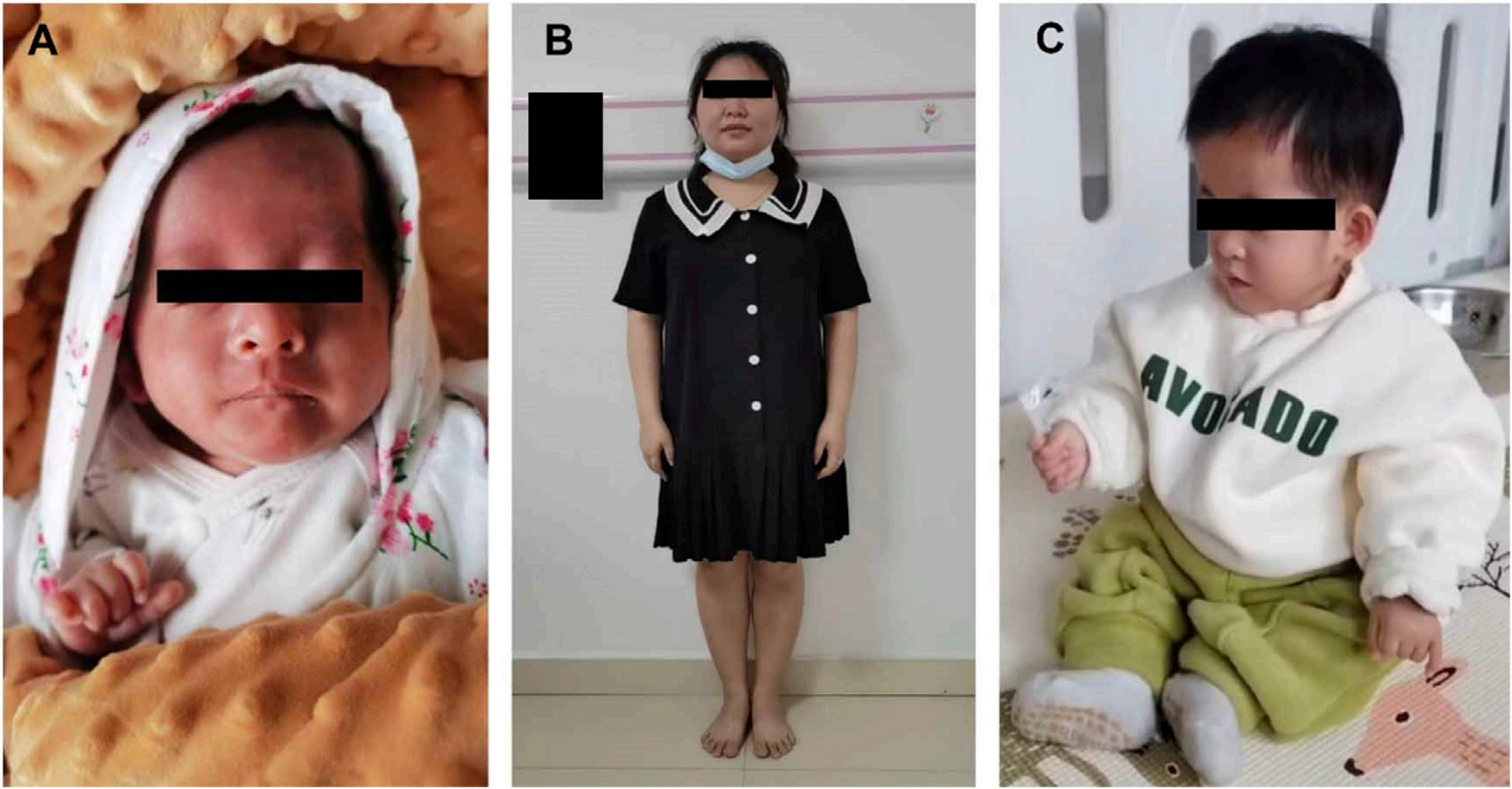
**(A)** Appearances of the infant at corrected gestational age of 6 months; **(B)** Mother; **(C)** Appearances of the child at corrected gestational age of 15 months.

Amniocentesis was performed at 20 gestational weeks for prenatal diagnosis. Fetal CMA showed two repeats (arr [GRCh37]11p15.5p15.4 (2,480,224_3,196,694)×3 and arr [GRCh37]11p15.5p15.4 (3,813,457_4,222,358)×3) in 11p15.4–15.5 (Figure 2). The repeated segments were similar to that in the propositus, containing *CDKN1C*, *KCNQ1*, and *KCNQ1OT1* imprinted genes, also known as imprinting center region 2 (IC2). MS-MLPA showed that, in the fetus, IC1 was normal, while IC2 had a high copy number of 3, and the methylation was enhanced with an average ratio of 0.69 (Figure 3). In the mother, IC1 was normal, IC2 had a high copy number of 3, and the methylation was low with an average ratio of 0.33 (Figure 4). In the grandfather, IC1 was normal, while IC2 had a high copy number of 3, and the methylation was low with an average ratio of 0.34 (Figure 5). The repeated segments (IC2) in 11p15.4–15.5 were almost identical in the fetus, mother, and grandfather. However, the methylation of IC2 in the fetus was higher than that in the mother and grandfather. After prenatal consultation, the woman chose to preserve the fetus. At 32 gestational weeks, fetal ultrasound showed biparietal diameter (BPD) 75 mm, femur length (FL) 46 mm, humerus length (HL) 51 mm, with an estimated GA of 27+4 weeks, indicating FGR. At 33 gestational weeks, the woman experienced acute loss of amniotic fluid with abnormal fetal cord blood flow. The infant was born on an emergent cesarean section with a birth weight of 950 g (<P3), and admitted to the neonatal intensive care unit (NICU). Standardized follow-up was performed in the outpatient department after discharge. At corrected GA of 6 months, the infant presented with forehead protrusion and slight facial asymmetry (Figure 6A). He had feeding difficulties and experienced extrauterine growth retardation. At corrected GA of 9 months, the body weight was 5,130 g (−4.2SD) and the height was 61 cm (−4.8SD). At corrected GA of 15 months, the body weight of 6,000 g (−4.8SD), height 67 cm (−5.3SD), and head circumference was 42 cm (−4.0SD). The motor development was normal (Figure 6C). The phenotype was quite consistent with SRS. Since short stature, the infant was treated with weekly injection of long-acting GH at a dose of 0.2 mg/kg.

## 5 Discussion

LOM of IC2, as both paternal and maternal alleles are unmethylated, with inactivation of *CDKN1C*, has been observed in 50% of BWS, (Brioude et al., 2018). However, IC2 alterations are rare in SRS. In the previous study, Mio C et al. reported a case of a girl with a mild SRS phenotype associated with a paternally inherited 1.4 kb deletion of IC2 (Mio et al., 2021). In the current study, we presented a SRS phenotype due to maternal IC2 repeats and methylation. *CDKN1C* located on IC2 is a cyclin-dependent kinase inhibitor that regulates cell proliferation and promotes cell cycle arrest. Normally, IC2 is methylated on the maternal allele *KCNQ1OT1*, thus *KCNQ1OT1* is repressed, allowing the expression *CDKN1C* (Chang and Bartolomei, 2020). Once maternal IC2 was activated by repeats or missense mutation, loss of *CDKN1C* expression might occur, contributing to SRS (Binder et al., 2020). CNV of maternal 11p15 accounts for only about 1% of the molecular pathogenic causes of SRS (Ishida, 2016). In the present case, the duplication of 11p15.4–15.5 mainly contained repeats of IC2 and enhanced maternal IC2 methylation. Considering the physiological and pathological effects of IC2, combined with the typical clinical manifestations (FGR, feeding difficulties, postnatal growth retardation, and distinctive features such as forehead protrusion and facial asymmetry), SRS was diagnosed. Although the mother and grandfather had the same CNV of 11p15, as for the mother, IC2 was paternal hypomethylated, similar to the physiological state, resulting in normal phenotypes. FGR and 11p15.4–15.5 duplication were found in two successive fetuses in this woman, and the location and length of the duplications were basically the same in both fetuses, indicating the conservative inheritance of imprinted genes. Meanwhile, the methylation of IC2 only increased in the maternal origin while remaining physiologically low in the paternal origin, indicating the epigenetic characteristics of imprinted genes during inheritance. It can be inferred that the woman has a 50% chance of passing 11p15 duplications carrying repeated IC2 with enhanced methylation to her offspring in each pregnancy. Through literature review, we learned that SRS caused by maternal 11p15 duplication containing IC2 is rare.Boonen SE reported maternally inherited duplication of 0.88 Mb including the *CDKN1C* gene causing intra-uterine growth restriction and postnatal short stature (Boonen et al., 2016). In some SRS cases, manifestations lasted for a short time, eased with age, and had a good prognosis (Hamza et al., 2023). Prenatal molecular diagnosis including detection of methylation levels at IC1 and IC2, as well as CNV at 11p15.5 can improve the diagnosis rate of affected individuals, enrich the genetic spectrum of the disease, contribute to incidence data, and enable growth trajectory monitoring and timely interventions for these patients early in life.

Follow-up and interventions for SRS need to be tailored according to specific manifestations at different stages. Most children with SRS have poor appetite and feeding difficulties during infancy, accompanied by gastroesophageal reflux and esophagitis (Blissett et al., 2001). Dietary adjustments, oral training, enteral feeding, and nutrient supplementation can be considered to increase infants’ appetite and ensure adequate calorie intake to prevent malnutrition. Most feeding problems ease with age. Those who have not completed catch-up growth by the age of 2 years should be closely monitored for physical development indicators, and growth hormone therapy should be initiated at the age of 4 years for those whose height is < −2.5 SD, on the basis of adequate caloric intake (Lee et al., 2003). Growth hormone therapy can significantly improve height in children with SRS (Chang and Bartolomei, 2020). In addition to nutrition and height, attention should also be paid to osteoporosis, obesity, type 2 diabetes, hypertension, and other diseases, as well as the social and psychological health of these children (Toumba et al., 2010).

## Data Availability

The original contributions presented in the study are publicly available. This data can be found here: https://www.ncbi.nlm.nih.gov/geo/query/acc.cgi?acc=GSE283758.
